# Bellevue Hospital—Notes of Practice

**Published:** 1876-05

**Authors:** 


Bellevue Hospital—Notes of Practice and Peculiarities of Treatment.—*Infantile Paralysis.*—These cases were interesting simply from the fact that there was so slight constitutional disturbance after the attack. One baby, twenty-two months old, born of healthy parents, but itself rather delicate, was put in bed at night five weeks ago, and the next morning was found unable to either sit or stand. The child was a trifle feverish for a few days only. The left leg had been restored, but the right exhibited a marked degree of motor paralysis. The temperature of the paralyzed leg was 3° F. less than the other. The other baby, eighteen months old, born of healthy parents, and itself always healthy, was placed in bed at night four weeks ago, and the morning following was found unable to stand, but the paralysis affected the left leg only. There was about the same difference in temperature between the right and left leg as in the other case, but the paralysis was more marked. The constitutional disturbance which followed the attack in this case was so slight, if there was any, that it was not noticed.

*Certain Peculiarities of a Diseased Ankle-joint.*—The case illustrated how much damage may follow a very slight injury of a joint, in the event that it is not properly treated after the occurrence of the accident. A man, æt. 40, hale and healthy, and weighing 270 pounds, "sprained his ankle" about two years ago; but the injury was so slight that it passed almost unnoticed, and gave him no great inconvenience, except when over-exercised, or slightly twisted, etc. As time passed along, the joint began to enlarge, and at the time of the examination it was seventeen inches in circumference, the extra size being produced by the inflammatory deposits which had been thrown out, simply because the subject was a vigorous, healthy man; whereas, in a weakly, broken-down constitution, the same amount of disturbance would probably have terminated in caries. The disease in this case was undoubtedly of traumatic origin. The instant the joint surfaces were crowded together, the patient complained of pain. The point in the treatment, therefore, was to relieve these surfaces of all pressure, which was accomplished by means of the splint commonly used by Dr. Sayre in the management of this class of cases.

*Temporary attacks of Aphasia—Loss of Power in the Right Arm.*—This case was worthy of note for the two items indicated.

A man, æt. sixty years, had two slight attacks of pleurisy three or four years ago, but aside from these had always been healthy until about two years ago, when he began to suffer more or less pain over the region of the sternum, and has also suffered somewhat from shortness of breath, especially on going up stairs. A few days ago, while sharpening his razor, he suddenly discovered that he was unable to speak, and about fifteen minutes elapsed before his power of speech returned. It was difficult to determine whether he could move his tongue or not at the time of the loss of ability to speak. There was marked loss of power in the right arm, but the leg remained unaffected. This attack was one of quite a number from which he has suffered. About a year ago he had a similar attack, which occurred about four o'clock in the morning. At that time he was unable to speak, but was able to move the tongue; his face was slightly drawn to the left side, but there was no trouble with the leg. The inability to speak soon passed away. Six weeks afterwards he had another attack while walking across the street, became paraplegic, the right arm became very much crippled, and he was also unable to speak. This attack lasted about two hours, and then passed away. His last attack has been described. The man did not suffer from headache, protruded the tongue correctly, and had the slight loss of power in the right arm already mentioned. Sensation was unimpaired. It was believed that these attacks were due to embolism. The man had valvular disease of the heart.

*Valuable point in Physical Diagnosis.*—The point relates to pulmonary resonance over the sternum. The edge of the right lung reaches to the *centre* of the sternum, as can be determined by *light* percussion. When the person is made to take a full inspiration, and hold it, pulmonary resonance can be easily detected entirely across the sternum. Some of the books teach that dulness upon percussion is normal over the sternum, hence the practical inference that when resonance is obtained over this region some abnormal condition is present, which is not necessarily true.

*Injection of Stimulants into the Trachea by means of the hypodermic Syringe.*—The patient was in a condition of collapse. About one drachm of a fluid, consisting of equal parts of water and brandy, was thrown slowly into the trachea by means of a hypodermic syringe, the needle of which had been thrust through its walls. The patient did not rally, but no ill effects were produced, nor inconvenience given.

The inner surface of the trachea is said to absorb volatile substances more rapidly than any other surface in the body.

*Item of interest in a case of Chronic Heart Disease.*—A noticeable feature of this case was the presence of well-developed valvular disease and hypertrophy with dilatation, without any history of rheumatism or syphilis, in a young man, aged 23 years.

The case, in addition, illustrated the occurrence of Bright's disease secondary to cardiac disease, and at the time of the examination he was also suffering from pleurisy with effusion.

*New idea relating to Chancroids and Stricture of the Rectum.* The interest in two cases which were seen centered upon the fact that they were illustrations (1) of the ease with which chancroids inoculate themselves, and (2) that stricture of the rectum may be a final result. Both patients were females. One showed chancroids near the meatus urinarius, in the fourchette, upon hemorrhoidal growths which were present around the verge of the anus, and the effects of the virus were plainly seen within the cavity of the rectum. When the rectum is reached troublesome ulceration becomes developed, and as a result of this stricture, which was well illustrated in the second patient.

They were regarded as more typical cases than are usually seen. These strictures are often spoken of as syphilitic strictures of the rectum, but it was believed that, in a very great majority of cases, they are of *chancroidal* origin. In a large percentage of cases at least there is no constitutional syphilis present, or evidence of its previous existence. The patient with stricture before us gave no history of constitutional syphilis, but there were well-marked chancroidal cicatrices present and a history to sustain them.

Something, it was said, could be done to prevent the spread of the disease up the rectum, and in this case nitric acid was applied, and the ulcers subsequently dressed with idoform. Such strictures, for obvious reasons, are not of common occurrence in the male subject.

*Hot Water as an agent for bringing about Relaxation of Contracted Muscles.*—The case illustrated in a striking manner the effect which is sometimes produced upon contracted muscles by the use of hot water. The water was poured upon the contracted parts for a considerable length of time at each sitting, and repeated twice a day.

When this measure was first resorted to in the case before us, the knees were held forcibly in contact with each other, and the heels were in close contact with the nates.

The man is now able, at the end of little more than one year, to separate the knees about eighteen inches, and can sit in a chair with his feet upon the floor. This relaxation is more than temporary, for the reason that it continues beyond the time in which the muscles are under the immediate influence of the hot water.—*Medical Record.*

